# Risk of cardiovascular events following COVID-19 in people with and without pre-existing chronic respiratory disease

**DOI:** 10.1093/ije/dyae068

**Published:** 2024-06-07

**Authors:** Hannah Whittaker, Constantinos Kallis, Thomas Bolton, Angela Wood, Samantha Walker, Aziz Sheikh, Alex Brownrigg, Ashley Akbari, Kamil Sterniczuk, Jennifer K Quint, CVD-COVID-UK/COVID-IMPACT Consortium

**Affiliations:** Respiratory EHR, School of Public Health, Imperial College London, London, UK; Respiratory EHR, School of Public Health, Imperial College London, London, UK; British Heart Foundation Data Science Centre, Health Data Research UK, London, UK; Department of Public Health and Primary Care, University of Cambridge, Cambridge, UK; Asthma + Lung, London, UK; British Heart Foundation Data Science Centre, Health Data Research UK, London, UK; British Heart Foundation Cardiovascular Epidemiology Unit, University of Cambridge, Cambridge, UK; Department of Public Health and Primary Care, University of Cambridge, Cambridge, UK; Asthma + Lung, London, UK; Usher Institute, University of Edinburgh, Edinburgh, UK; BREATHE, Health Data Research UK, London, UK; Population Data Science, Swansea University Medical School, Swansea University, Swansea, UK; BREATHE, Health Data Research UK, London, UK; Respiratory EHR, School of Public Health, Imperial College London, London, UK

**Keywords:** COVID-19, COPD, asthma, cardiovascular disease

## Abstract

**Background:**

COVID-19 is associated with cardiovascular outcomes in the general population, but it is unknown whether people with chronic respiratory disease (CRD) have a higher risk of cardiovascular events post-COVID-19 compared with the general population and, if so, what respiratory-related factors may modify this risk in these people.

**Methods:**

Primary and secondary care data from the National Health Service England were used to define a population of adults in England with COVID-19 (index date) between 1 January 2020 and 30 November 2021. Adjusted Cox proportional hazard regression was used to quantify the association between CRD, asthma-related factors, chronic obstructive pulmonary disease (COPD)-related factors, and risk of cardiovascular events. Asthma-specific factors included baseline asthma control, exacerbations, and inhaled corticosteroid (ICS) dose. COPD-specific risk factors included baseline ICS and exacerbations. Secondary objectives quantified the impact of COVID-19 hospitalisation and vaccine dose on cardiovascular outcomes.

**Results:**

Of 3 670 455 people, those with CRD had a higher risk of cardiovascular events [adjusted hazard ratio (HR_adj_), 1.08; 95% confidence interval (CI) 1.06–1.11], heart failure (HR_adj_, 1.17; 95% CI, 1.12–1.22), angina (HR_adj_, 1.13; 95% CI, 1.06–1.20) and pulmonary emboli (HR_adj_, 1.24; 95% CI, 1.15–1.33) compared with people without CRD. In people with asthma or COPD, baseline exacerbations were associated with a higher risk of cardiovascular outcomes (HR_adj_, 1.36; 95% CI, 1.27–1.00 and HR_adj_, 1.35; 95% CI, 1.24–1.46, respectively). Regardless of CRD, the risk of cardiovascular events was lower with increasing COVID-19 vaccine dose.

**Conclusions:**

Higher risk of cardiovascular events post-COVID-19 might be explained by the underlying severity of the CRD, and COVID-19 vaccines were beneficial to both people with and those without CRD with regards to cardiovascualr events.

Key Messages

- We investigated whether pre-existing chronic respiratory disease was associated with cardiovascular outcomes following COVID-19.
- We found that chronic respiratory disease and chronic respiratory disease severity were associated with a higher risk of cardiovascular outcomes following COVID-19. However, this is likely due to the underlying chronic respiratory disease rather than COVID-19 itself.
- Improved management of chronic respiratory diseases is necessary to help to reduce the risk of cardiovascular disease in these populations.

## Introduction

The SARS-CoV-2 virus induces a proinflammatory state, increasing the risk of developing further conditions including chronic and acute cardiovascular events.^1–6^ Those with chronic respiratory disease (CRD) are at a higher risk of cardiovascular disease than the general population.^7^^,^^8^ Cardiovascular disease is a common comorbidity in people with chronic obstructive pulmonary disease (COPD), asthma, interstitial pulmonary fibrosis, and bronchiectasis^7^^,^^9–15^and people with cystic fibrosis have a high prevalence of risk factors for cardiovascular disease.^16^

Risk of cardiovascular events increases in people with COPD, particularly following an acute pulmonary infection such as an exacerbation of COPD.^17^ It is possible that people with CRD have a different risk of developing cardiovascular outcomes following an infection, such as COVID-19, compared with people without CRD. In addition, the association between COVID-19 vaccination and the reduced risk of cardiovascular events remains in people with and without CRD separately, and to what extent this risk differs between those with and without CRD, given that people with CRD already have a different baseline level of inflammation, is unknown.^6^^,^^7^^,^^18^

Using routinely collected electronic health care data in England, we investigated the association between pre-existing CRD and cardiovascular outcomes post-COVID-19 in a population of people with COVID-19, and the association between markers of asthma and COPD severity and risk of cardiovascular events post-COVID-19, given that asthma and COPD are leading causes of CRD worldwide.^9^

## Methods

### Study population

De-identified data were accessed and analysed through the British Heart Foundation Data Science Centre CVD-COVID-UK/COVID-IMPACT consortium within the National Health Service (NHS) England secure, privacy-protecting Secure Data Environment (SDE) service for England.^19^ The data included: primary care data, hospital data (inpatient, outpatient, emergency department and critical care), mortality data (causes of death from death certificates), COVID-19 laboratory test data, COVID-19 vaccine data and data on dispensed medications (Supplementary Table S1, available as Supplementary data at *IJE* online). All code lists for variables used in this study can be found at [https://github.com/BHFDSC/CCU035_01/blob/main/phenotypes/CCU035_01-D01-codelist.py].

Primary care data from General Practice Extraction Service for Pandemic Planning and Research (GDPPR) included over 56 million people registered at a general practice (GP) in England who were alive on 30 November 2021. We defined a population of people aged >18 years diagnosed with COVID-19 from 1 January 2020. COVID-19 diagnosis (index date) was determined through primary or secondary care and SARS-CoV-2 laboratory test data. People were excluded if they had a COVID-19 vaccination before their first COVID-19 diagnosis. People were followed up until 30 November 2021, or earlier at the individual’s second COVID-19 diagnosis date, first COVID-19 vaccination date or date at which they died (Supplementary Figure S1, Supplementary Material Page 2, available as Supplementary data at *IJE* online). Pre-existing CRD was defined as a primary care diagnosis of asthma, COPD, bronchiectasis, cystic fibrosis, or pulmonary fibrosis prior to index date, using SNOMED CT codes.

### Asthma cohort

From this population, a cohort of people with pre-existing asthma was defined. Asthma-related exposures included: asthma exacerbations, asthma control and ICS dose. Asthma exacerbations were defined as one or more of: (i) course of oral corticosteroids; (ii) hospital admission for an asthma exacerbation (using the International Classification Of Diseases, Revision 10 (ICD-10) codes in any position); and (iii) emergency department visit for an asthma-related cause. Asthma control was defined as one or more of: (i) two or more courses of oral corticosteroids or two or more hospital admission or accident and emergency attendances in the year prior to index; (ii) 6 months or more of short-acting beta agonist (SABA) use in the year prior to index date. ICS dose, based on the global initiative for asthma (GINA) guidelines, was categorized as low, medium or high, using the last ICS prescription in the year prior to index date.^20^ GitHub link to algorithm used was [https://github.com/BHFDSC/CCU035_01/tree/main/code/CCU035_01-D30-ics_24h].

### COPD cohort

From the base population, a cohort of people with pre-existing COPD was defined. COPD-specific exposures included ICS use and COPD exacerbations. ICS (users vs non-users) was defined as having one or more prescriptions of ICS in three-quarters of the year prior to index date.^21^ COPD exacerbations were defined as one or more exacerbations requiring hospitalization or a prescription of respiratory-related antibiotics and oral corticosteroids for 5–14 days.^22^^,^^23^

### Cardiovascular outcomes

Cardiovascular outcomes over follow-up were defined using primary, secondary and mortality data. First cardiovascular event during follow-up was identified. Outcomes were defined as:

- venous thromboembolism (VTE) events (deep vein thrombosis and portal vein thrombosis);
- coagulopathy events (disseminated intravascular coagulopathy, thrombotic thrombocytopenic purpura, thrombocytopenia, and thrombophilia);
- heart failure (HF), including cardiomyopathy;
- angina (stable and unstable angina);
- myocarditis and pericarditis;
- stroke (ischaemic and haemorrhagic);
- myocardial infarction (MI);
- pulmonary embolism; and
- arrhythmias.

A composite cardiovascular outcome was also defined as a combination of all groups of cardiovascular events. For the asthma and COPD cohorts, these outcomes events were categorized into four variables: composite, arterial, venous and other events (Supplementary Material Page 2, available as Supplementary data at *IJE* online).

### COVID-19 vaccination

COVID-19 vaccination data were extracted from the National Immunisation Management System COVID-19 vaccination database. Dose number was calculated based on COVID-19 vaccination date and duplicate dates were removed.

### Statistical analyses

Analyses were performed according to a pre-specified analysis plan published on GitHub, along with the phenotyping and analysis code [https://github.com/BHFDSC/CCU035_01]. Cox proportional hazard regression was used to quantify the association between CRD and risk of cardiovascular outcomes post-COVID-19. Standard error estimates were adjusted for clustering of people in GPs as it was possible the treatment of people with COVID-19 differed by GPs across England. Cox regression was also used to investigate the association between; (i) asthma or COPD and post-COVID-19 cardiovascular events in the base population; (ii) asthma-related factors and post-COVID-19 cardiovascular events in the asthma cohort; and (iii) COPD-related factors and post-COVID-19 cardiovascular events in the COPD cohort. Models were adjusted for patient demographics, comorbidities and disease-specific confounders (Supplementary Material Page 1, Supplementary Figure S2, available as Supplementary data at *IJE* online). Missing data were reported, and complete case analysis on all covariates was used. Supplementary Figure S2 (available as Supplementary data at *IJE* online) illustrates the choice of covariates used in analyses.

Secondary analyses included the following:

- effect modification by COVID-19 severity determined by whether people were hospitalized for COVID-19 within 2 weeks of their COVID-19 diagnosis;
- effect modification by vaccination status, whereby follow-up time was split into the unvaccinated period (reference category), the period from first to second vaccine, the period from second to third vaccine (booster vaccination) and the period from the third vaccine to the end of follow-up. All types of COVID-19 vaccines were used. In this analysis, end of follow-up was defined as 30 November 2021, second COVID-19 diagnosis or death. First cardiovascular event over follow-up was identified.

A sensitivity analysis was performed whereby people with a history of acute cardiovascular outcomes (venous thrombosis, dissected artery and stroke) events prior to COVID-19 diagnosis were excluded. A further sensitivity analysis censored follow-up time at 30 days to determine the acute risk of cardiovascular outcomes post-COVID-19. Due to multiple testing, Bonferroni correction was applied (Supplementary Material Page 2, available as Supplementary data at *IJE* online). To ensure anonymity and compliance with the CVD-COVID-UK consortium, all reported numbers were rounded to the nearest 5. Counts less than 10 were expressed as ‘<10’.

## Results

**Figure 1.**
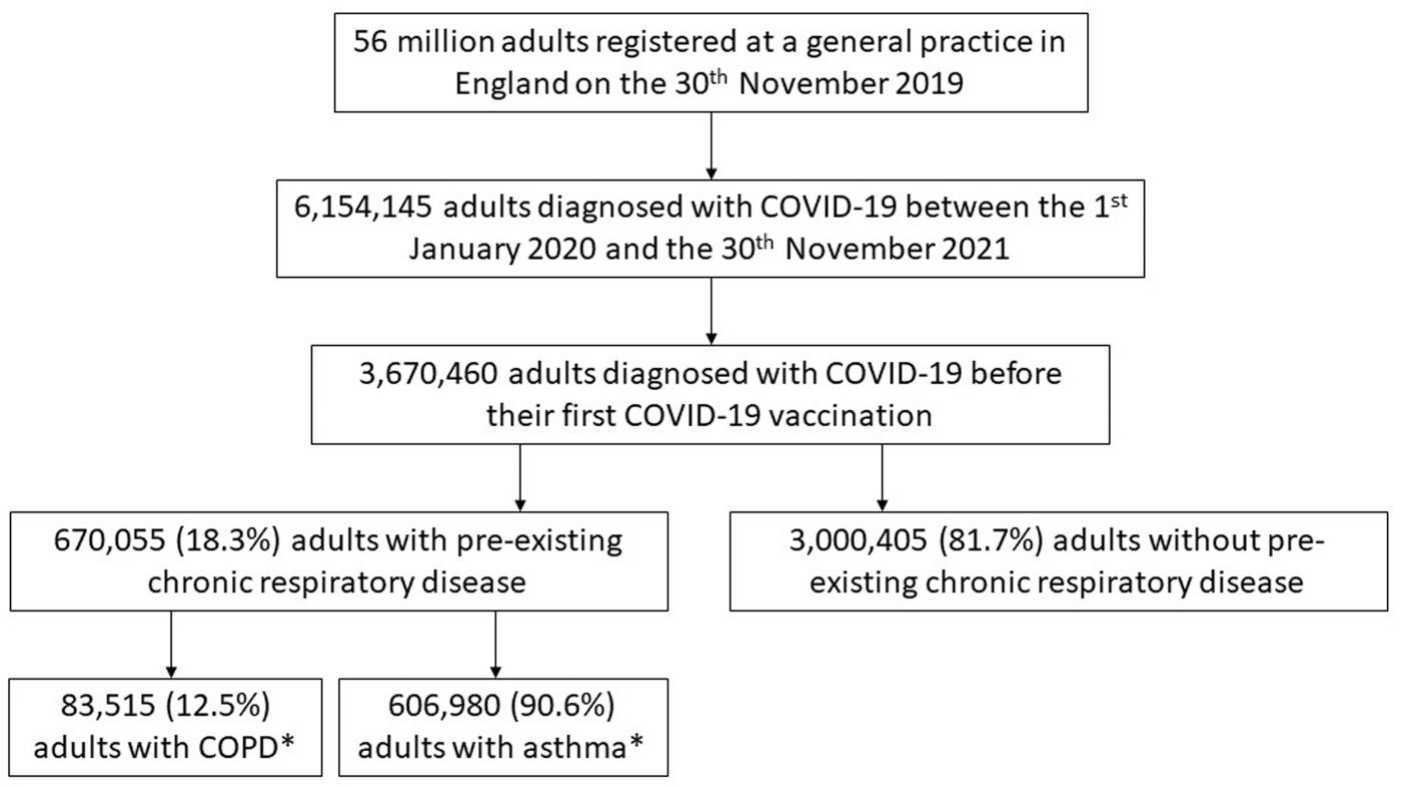
Flow diagram illustrating inclusion of study population. Numbers rounded to the nearest 5. *People with pre-existing COPD and asthma were not mutually exclusive

In all, 3 670 460 people were diagnosed with COVID-19 between 1 January 2020 and 30 November 2021 prior to their first COVID-19 vaccination. Of these, 670 055 (18.3%) had CRD (Figure 1); 606 980 (16.5%) had asthma; 83 515 (2.3%) had COPD; 17 095 (0.5%) had bronchiectasis; 8980 (0.2%) had pulmonary fibrosis; and 715 (0.02%) had cystic fibrosis. CRD groups were not mutually exclusive. Median follow-up from COVID-19 diagnosis to end of follow-up was 3.9 months [interquartile range (IQR), 1.9–6.6 months]; 43 820 (1.2%) people had a recorded cardiovascular event during the follow-up (Supplementary Table S2, available as Supplementary data at *IJE* online).

People with CRD were more likely to be ex-smokers, less likely to live in London, more likely to be obese, have hypertension or chronic kidney disease, and to have cardiovascular disease prior to COVID-19 diagnosis (Table 1). Individuals included in our study were slightly younger and less deprived but were similar in terms of all other characteristics (Supplementary Table S3, available as Supplementary data at *IJE* online).

**Table 1.** Baseline characteristics of people with and without pre-existing chronic respiratory disease

Baseline covariate	Total cohort (*n *=* *3 670 460)	No pre-existing respiratory disease (*n *=* *3 000 405)	Pre-existing respiratory disease (*n *=* *670 055)
Age in years, mean (SD)	43.1 (18.4)	42.7 (18.1)	44.6 (19.8)
Female sex	1 982 115 (54.0)	1 618 085 (53.9)	364 030 (54.3)
Smoking status			
Non-smoker	2 150 610 (58.6)	1 783 250 (59.4)	367 355 (54.8)
Ex-smoker	833 550 (22.7)	644 235 (21.5)	189 315 (28.3)
Current smoker	534 220 (14.6)	429 270 (14.3)	104 950 (15.7)
Missing	152 075 (4.1)	143 650 (4.8)	8 430 (1.3)
IMD			
1 (most deprived)	468 930 (14.8	376 165 (12.5	92 770 (13.9
2	455 030 (12.4)	371 090 (12.4)	83 940 (12.5)
3	433 325 (11.8)	355 485 (11.9)	77 840 (11.6)
4	391 800 (10.7)	321 870 (10.7)	69 930 (10.4)
5	363 685 (9.9)	97 990 (9.9)	65 695 (9.8)
6	341 140 (9.3)	279 875 (9.3)	61 265 (9.1)
7	325 870 (8.9)	266 585 (8.9)	59 285 (8.9)
8	317 555 (8.7)	259 965 (8.7)	57 590 (8.6)
9	296 420 (8.1)	242 775 (8.1)	53 645 (8.0)
10 (least deprived)	260 590 (7.1)	213 640 (7.1)	46 950 (7.0)
Missing	16 115 (0.4)	14 970 (0.5)	1 145 (0.2)
Region			
Southeast	455 290 (12.4)	373 910 (12.5)	81 380 (12.2)
Southwest	181 690 (5.0)	146 180 (4.9)	35 515 (5.3)
London	638 670 (17.4)	543 805 (18.1)	94 866 (14.2)
East Midlands	269 940 (7.4	219 730 (7.3)	50 210 (7.5)
West Midlands	477 860 (13.0)	386 100 (12.9)	91 760 (13.7)
East of England	305 840 (8.3	250 270 (8.3)	55 570 (8.3)
Yorkshire and the Humber	394 475 (10.8)	318 510 (10.6)	75 964 (11.3)
Northeast	162 560 (4.4)	128 925 (4.3)	33 640 (5.0)
Northwest	580 275 (15.8)	465 435 (15.5)	114 840 (17.1)
Missing	203 855 (5.6)	167 545 (5.6)	36 310 (5.4)
BMI			
Underweight	120 075 (3.3)	96 640 (3.2)	23 435 (3.5)
Normal	902 700 (24.6)	743 060 (24.8)	159 640 (23.8)
Overweight	791 070 (21.6)	645 145 (21.5)	145 925 (21.8)
Obese	1 417 310 (38.6)	1 119 250 (37.3)	298 060 (44.5)
Missing	439 305 (12.0)	396 310 (13.2)	42 995 (6.4)
Hypertension	754 915 (20.6)	582 365 (19.4)	172 550 (25.8)
Diabetes	320 765 (8.7)	241 460 (8.1)	79 305 (11.8)
Liver disease	12 185 (0.3)	8 855 (0.3)	3 330 (0.50)
Chronic kidney disease	252 660 (6.9)	185 680 (6.2)	66 980 (10.0)
Dementia	101 130 (2.8)	78 870 (2.6)	22 260 (3.3)
Previous severe cardiovascular events	344 445 (9.4)	250 725 (8.4)	93 725 (14.0)
Cardiovascular-related medication	797 235 (21.7)	614 915 (20.5)	182 322 (27.2)

Missing data were reported for IMD, region and BMI only.

BMI, body mass index; IMD, index of multiple deprivation; SD, standard deviation.

### CRD and risk of post-COVID-19 cardiovascular events

**Figure 2.**
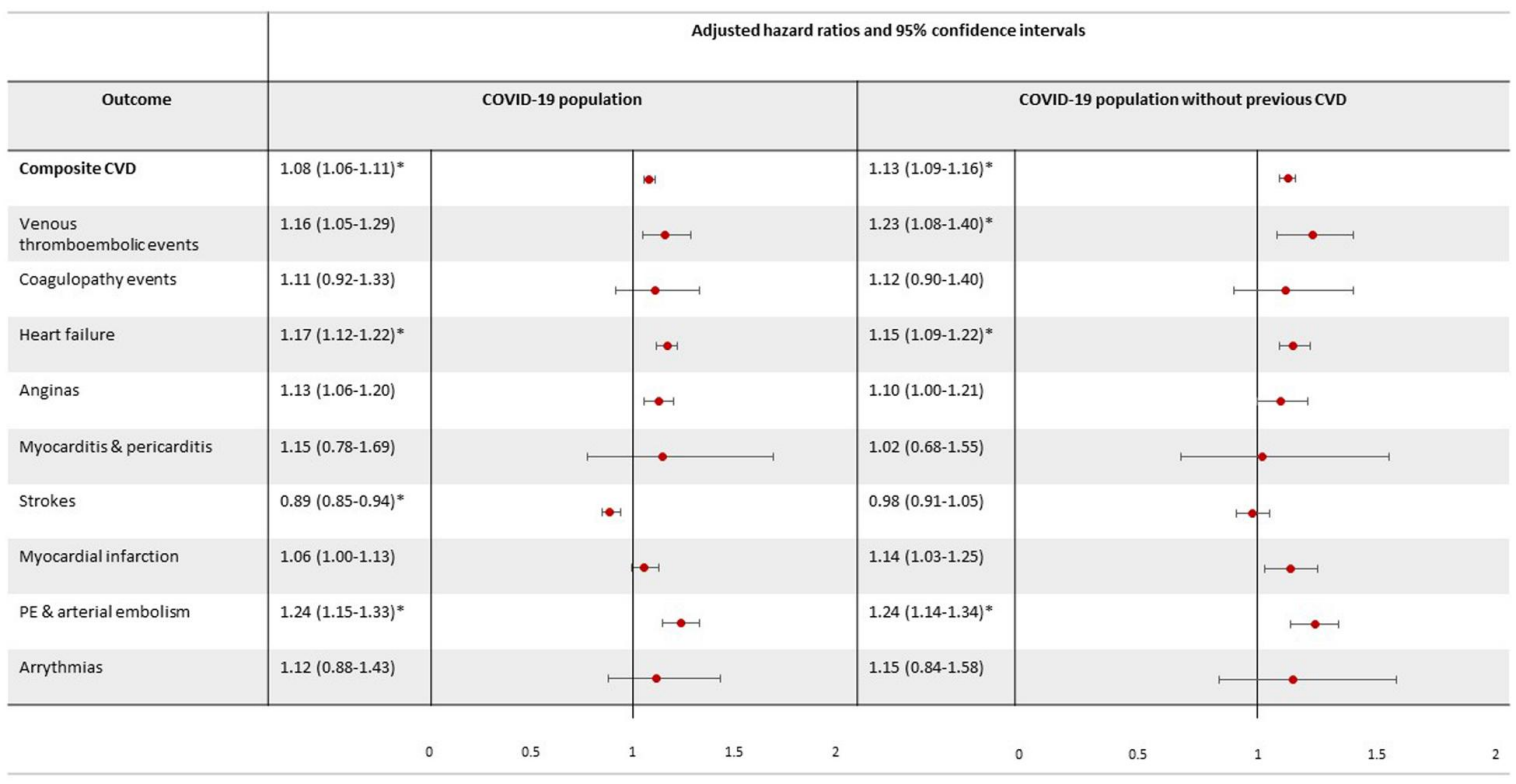
Risk of post-COVID-19 cardiovascular outcomes (and each cardiovascular component) in people with pre-existing chronic respiratory disease compared with people without pre-existing chronic respiratory disease. Venous thromboembolic events include deep vein and portal vein thrombosis. Coagulopathy events include thrombocytopenia, thrombophilia and mesenteric thrombus, pulmonary embolism (PE). Risk of cardiovascular outcomes is reported in all people with COVID-19 and people with no history of cardiovascular events prior to COVID-19 separately. *P < 0.005

People with CRD had a higher risk of composite cardiovascular outcomes [adjusted hazard ratio (HR_adj_), 1.08; 95% confidence interval (CI) 1.06–1.11], HF (HR_adj_, 1.17; 95% CI, 1.12–1.22) and pulmonary embolism (HR_adj_, 1.24; 95% CI, 1.15–1.33) compared with those without CRD, and less likely to have a stroke compared with those without CRD (HR_adj_, 0.89; 95% CI, 0.85–0.94) (Figure 2, Supplementary Table S4, available as Supplementary data at *IJE* online).

After excluding people with a history of severe cardiovascular events, people with CRD had a higher risk of any cardiovascular event (HR_adj_, 1.13; 95% CI, 1.09–1.16), VTE (HR_adj_, 1.23; 95% CI, 1.08–1.40), HF (HR_adj_, 1.15; 95% CI, 1.09–1.22) and pulmonary embolism (HR_adj_, 1.24; 95% CI, 1.14–1.34) compared with people without CRD (Figure 2, Supplementary Table S5, available as Supplementary data at *IJE* online). After limiting follow-up to the first 30 days following COVID-19, 22 559 (51.5%) cardiovascular events occurred and results remained consistent; however, a higher risk of MI was seen in people with CRD compared with people without CRD (HR_adj_, 1.20; 95% CI, 1.0–1.35; Supplementary Table S6, available as Supplementary data at *IJE* online) and there was no association between pre-existing chronic respiratory disease and HF following COVID-19.

### COVID-19 severity and risk of post-COVID-19 cardiovascular outcomes

Of 3 670 460 people with COVID-19, 250 370 (6.8%) were hospitalized and 3 420 085 (93.2%) were not. In people who were not hospitalized for COVID-19, there was an association between CRD and a higher risk of composite cardiovascular events (HR_adj_, 1.11; 95% CI 1.08–1.14) including VTE, HF, angina and pulmonary embolisms. After excluding people with a history of severe cardiovascular events, CRD was associated with composite CVD, HF and pulmonary embolisms only. In people who were hospitalized for COVID-19, there was an association between CRD and decreased risk of stroke only (HR_adj_, 0.76; 95% CI, 0.70–0.82) (Supplementary Tables S7 and S8, available as Supplementary data at *IJE* online).

### COVID-19 vaccination and risk of post-COVID-19 cardiovascular outcomes

**Figure 3.**
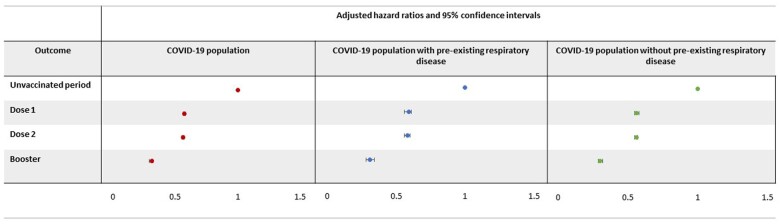
Association between increasing number of COVID-19 vaccinations and risk of cardiovascular outcomes in people who had been diagnosed with COVID-19. Reference period is the unvaccinated period from COVID-19 diagnosis to first COVID-19 vaccination or censoring. The Dose 1 period is from first COVID-19 vaccination to second COVID-19 vaccination or censoring. The Dose 2 period is from the second COVID-19 vaccination to booster or censoring. The booster period is from the booster vaccine to end of follow-up or censoring

A total of 2 652 210 (70.4%) people had at least one COVID-19 vaccine after their first COVID-19 diagnosis up until 30 November 2021. Of these people, 218 970 (8.3%) had one vaccine, 1 708 275 (64.4%) had two and 724 965 (27.3%) had two COVID-19 vaccines and the COVID-19 booster. Compared with the period between COVID-19 diagnosis and Dose 1 (i.e. the unvaccinated period), risk of composite cardiovascular outcomes was lower with each subsequent COVID-19 vaccine dose. There was no effect modification of CRD on the association between dose number and risk of cardiovascular outcomes (Figure 3, Supplementary Table S9, available as Supplementary data at *IJE* online).

### Asthma and risk of post-COVID-19 cardiovascular outcomes

People with pre-existing asthma had a higher risk of composite cardiovascular events as well as HF, angina and pulmonary emboli compared with people without asthma (HR_adj_, 1.05; 95% CI, 1.02–1.08; HR_adj_, 1.08; 95% CI, 1.04–1.13; HR_adj_, 1.11; 95% CI, 1.03–1.20; HR_adj_, 1.16; 95% CI, 1.07–1.26, respectively; Supplementary Table S10, available as Supplementary data at *IJE* online) and were less likely to have a stroke event (HR_adj_, 0.90; 95% CI, 0.86–0.95). After excluding patients with a history of severe cardiovascular events prior to COVID-19, pre-existing asthma was associated with an increased risk of composite cardiovascular events, VTE and pulmonary emboli only.

**Figure 4.**
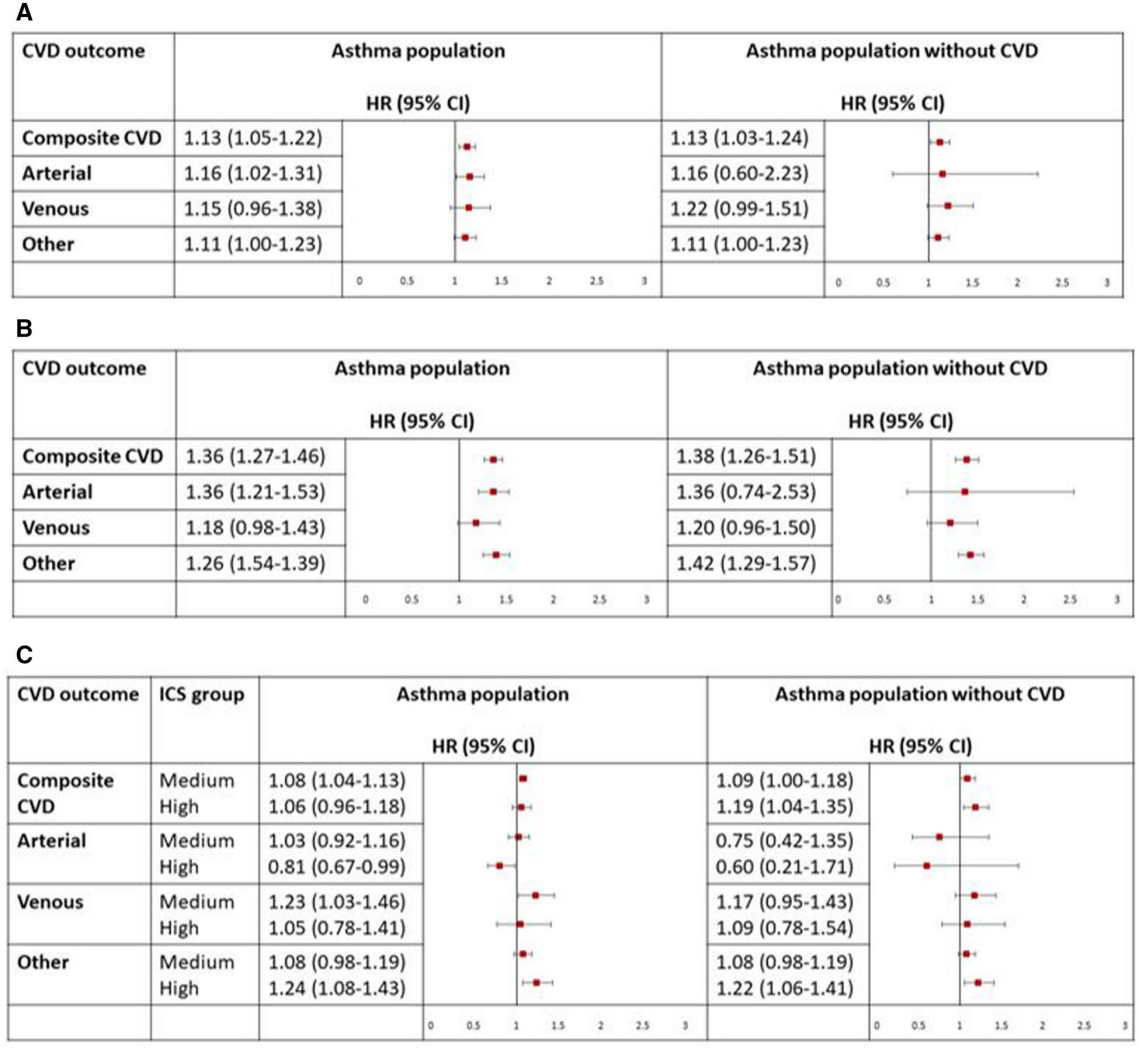
Risk of cardiovascular outcomes following COVID-19 by A) asthma control, B) asthma exacerbations and C) inhaled corticosteroid dose in people with asthma. Arterial events include disseminated intravascular coagulation-related events, stroke-related events and myocardial infarction. Venous events include venous thrombosis-related events and pulmonary embolism-related events. Other cardiovascular (CV) events include heart failure, angina, myocarditis, pericarditis, and arrythmias. Risk of CV events is reported in all patients with COVID-19 and asthma and asthma patients with no history of CV events prior to COVID-19 separately. Reference groups include: (A) those without poor asthma control, (B) those whose condition did not exacerbate, (C) low-dose inhaled corticosteroid. HR: Hazard Ratio; CI: Confidence Interval; CVD: Cardiovascular Disease

Of those with asthma, patients with better asthma control had a higher risk of composite cardiovascular outcomes compared with people with poorer asthma control (HR_adj_, 1.13; 95% CI, 1.05–1.22; Figure 4 and Supplementary Table S11, available as Supplementary data at *IJE* online). Those with one or more exacerbations at baseline had a higher risk of composite cardiovascular outcomes, arterial-related outcomes and other cardiovascular-related outcomes compared with people with asthma whose condition did not exacerbate at baseline (HR_adj_, 1.36; 95% CI, 1.27–1.46; HR_adj_, 1.36; 95% CI, 1.21–1.53; HR_adj_, 1.39; 95% CI, 1.26–1.54, respectively). Last, asthma patients on high-dose ICS had a higher risk of other cardiovascular-related events post-COVID-19 compared with those on low-dose ICS (HR_adj_, 1.24; 95% CI, 1.08–1.43).

### COPD and risk of post-COVID-19 cardiovascular outcomes

People with pre-existing COPD had an increased risk of cardiovascular outcomes as well as HF, angina and pulmonary emboli compared with those without COPD (HR_adj_, 1.11; 95% CI, 1.07–1.14; HR_adj_, 1.25; 95% CI, 1.18–1.31; HR_adj_, 1.14; 95% CI, 1.04–1.24; and HR_adj_, 1.36; 95% CI, 1.21–1.52, respectively; Supplementary Table S12, available as Supplementary data at *IJE* online) and a decreased risk of stroke compared with those without COPD (HR_adj_, 0.87; 95% CI, 0.81–0.93). After excluding patients with a history of severe cardiovascular events prior to COVID-19, a similar pattern of association was seen; however, there was no association between COPD and risk of stroke or angina.

**Figure 5.**
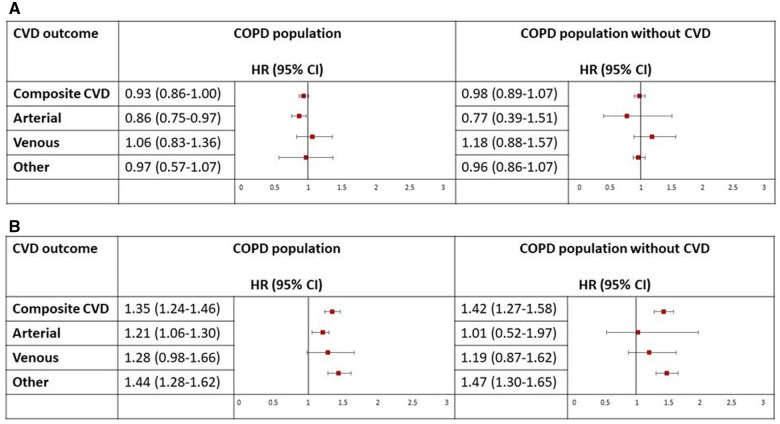
Risk of cardiovascular outcomes following COVID-19 by A) inhaled corticosteroid use and B) exacerbations in people with chronic obstructive pulmonary disease. Arterial events include disseminated intravascular coagulation-related events, stroke-related events and myocardial infarction. Venous events include venous thrombosis-related events and pulmonary embolism-related events. Other cardiovascular (CV) events include heart failure, angina, myocarditis, pericarditis and arrythmias. Risk of CV is reported in all patients with COVID-19 and COPD, and COPD patients with no history of CV events prior to COVID-19, separately. Reference groups include: (A) those not on inhaled corticosteroid and (B) those whose condition did not exacerbate. HR: Hazard Ratio; CI: Confidence Interval; CVD: Cardiovascular Disease; COPD: Chronic Obstructive Pulmonary Disease

Of those with COPD, no association between baseline ICS use and risk of cardiovascular outcomes post-COVID-19 was seen (Figure 5 and Supplementary Table S13, available as Supplementary data at *IJE* online). However, COPD patients whose condition exacerbated at baseline had a higher risk of composite cardiovascular events, specifically arterial and other cardiovascular-related events, post-COVID-19 compared with those whose condition did not exacerbate at baseline (HR_adj_, 1.35; 95% CI, 1.24–1.46; HR_adj_, 1.21; 95% CI, 1.06–1.39; and HR_adj_, 1.44; 95% CI, 1.28–1.62, respectively). The association seen for composite and other cardiovascular events remained consistent after excluding people with a history of severe cardiovascular events.

## Discussion

Using routinely collected data on over 3 million people with COVID-19, we found:

- CRD was associated with a higher risk of post-COVID-19 cardiovascular events including HF and pulmonary emboli;
- COVID-19 severity modified the association between CRD and risk of cardiovascular outcomes;
- the COVID-19 vaccine reduced the risk of any cardiovascular outcome in people who had been diagnosed with COVID-19 regardless of CRD;
- risk factors related to asthma or COPD disease severity were associated with an increased risk of post-COVID-19 cardiovascular events.

A previous study of 48 million people in England and Wales found that people with COVID-19 had a higher risk of arterial thrombosis and VTE compared with people without COVID-19, but the risk attenuated with time since COVID-19.^5^ Other studies have found that people hospitalized with COVID-19 had a higher risk of HF, stroke, ischemic heart disease and VTE compared with people who did not have COVID-19.^6^^,^^24^^,^^25^ Our study extends previous work by comparing these risks between subgroups of people with and without CRD. The higher risk of HF seen may be due the CRD rather than COVID-19, given that HF is a common comorbidity among people with CRD with overlapping symptoms.^7^^,^^26^^,^^27^ This might also be the case for the increased risk of pulmonary emboli. Studies have shown that risk of pulmonary embolus is higher in people with asthma and COPD compared with the general population, and is particularly higher in people with severe asthma compared with people with mild-to-moderate asthma and during or following an acute exacerbation of COPD.^28^^,^^29^ Pulmonary emboli and CRD also share common risk factors, including age and immobilization; however, the exact mechanism driving the association between pulmonary emboli and CRD is unclear.^30^ In addition, although we found that risk of MI following COVID-19 was not associated with CRD in our main analysis, we found that CRD was associated with a higher risk of MI within 30 days following COVID-19, as seen with pulmonary emboli. These results reinforce the fact that higher risk of pulmonary emboli following COVID-19 may be related to the underlying CRD rather than COVID-19; however, the higher risk of MI seen in people with CRD may be due to acute infection in these individuals. Previous studies show that risk of acute MI is highest in the first 27 weeks following COVID-19 and declines thereafter.^5^

In terms of the COVID-19 vaccine, our results were consistent with previous literature. Previous studies found that having one or more COVID-19 vaccinations post-COVID-19 decreased the rate of various long-covid symptoms including chest pain or tightness, palpitations and breathlessness.^6^ Similarly, other studies have found an association between the COVID-19 vaccine and a reduced risk of cardiovascular events, including MI and stroke, after SARS-CoV-2 infection.^18^ Surprisingly, we found that people with CRD who were hospitalized for COVID-19 had a lower risk of stroke compared with people without CRD, but similar risks of all other cardiovascular outcomes. It is possible that the level of COVID-19 severity has a larger impact on cardiovascular outcomes than CRD. The lower risk of stroke in people with CRD could be due to better monitoring of these people in hospital. It is well known that risk of ischaemic events increases after acute respiratory events, notably hospitalization due to acute respiratory events such as exacerbations.^17^ It is possible that care of people with CRD upon hospitalization with COVID-19 is better because CRD is a known risk factor for stoke.

Furthermore, we found that in people with asthma or COPD, markers of severe disease were associated with a higher risk of post-COVID-19 cardiovascular events. Post-hoc findings from COPD trials show an association between exacerbations and a higher risk of a subsequent cardiovascular events, which remained elevated for up to a year following an exacerbation.^31^^,^^32^ Therefore, it is likely that the association between exacerbations and risk of post-COVID-19 cardiovascular events in people with COPD is due to COPD severity. Risk factors related to age and airway obstruction have been shown to be strong predictors of cardiovascular disease in people with asthma, as well as COPD, and could explain the association seen between CRD severity and post-COVID-19 cardiovascular events.^33^ Despite this, our study found that irrespective of CRD, the COVID-19 vaccination reduces cardiovascular risk following COVID-19 infection. The COVID-19 vaccination should therefore be encouraged in all individuals, and people with CRD should be managed as robustly as possible to reduce the risk of cardiovascular events. Further studies investigating improved management of people with CRD, and notably severe CRD, should be explored to reduce the risk of future cardiovascular events following COVID-19.

Despite this study’s strengths, including large sample size, one limitation is the exclusion of people with a COVID-19 vaccination prior to their first COVID-19 diagnosis, which decreased number of individuals included in the study. Risk of post-COVID-19 outcomes and complications are known to be modified by the COVID-19 vaccine. In addition, we did not separate cardiovascular outcomes in the COVID-19 vaccine analysis, nor the COPD and asthma-specific analyses, due to low event numbers and reduced power. Results where a higher risk was seen for the composite cardiovascular outcome, but not in any cardiovascular subgroup, should be interpreted with caution for this reason. In addition, missing IMD, region and BMI data were reported and, due to complete case analysis, individuals with any of these missing data were not included in the fully adjusted analyses. Imputation was not performed as data are not missing at random, due to the nature of the data source. Despite this, our population size remained large. Confounding by indication in the ICS-related analyses could have led to associations seen for high-dose ICS or ICS use (in asthma and COPD cohorts, respectively) and results should be interpreted accordingly. Smoking status was determined as current, ex or never smokers, as pack-years of smoking were not available in the data. Last, spirometry data were not available and therefore were not used in the definition of COPD nor in the adjusted models and therefore, residual confounding may exist.

## Conclusion

Using data on over 3 million people with COVID-19 in England, we found that people with CRD had a modest higher risk of HF and pulmonary emboli compared with people without CRD, but the risk might be explained at least in part by the underlying respiratory condition and severity of that condition. COVID-19 vaccination reduced the risk of cardiovascular events following COVID-19, regardless of whether people had CRD.

## Ethics approval

The CVD-COVID-UK/COVID-IMPACT programme led by the BHF Data Science Centre [https://bhfdatasciencecentre.org/] received approval to access data in NHS England’s Secure Data Environment (SDE) service for England from the Independent Group Advising on the Release of Data (IGARD) [https://digital.nhs.uk/about-nhs-digital/corporate-information-and-documents/independent-group-advising-on-the-release-of-data] via an application made in the Data Access Request Service (DARS) Online system (ref. DARS-NIC-381078-Y9C5K) [https://digital.nhs.uk/services/data-access-request-service-dars/dars-products-and-services]. The CVD-COVID-UK/COVID-IMPACT Approvals and Oversight Board [https://bhfdatasciencecentre.org/areas/cvd-covid-uk-covid-impact/] subsequently granted approval to this project to access the data within NHS England’s SDE service for England. The de-identified data used in this study were made available to accredited researchers only. Those wishing to gain access to the data should contact [bhfdsc@hdruk.ac.uk] in the first instance. Patients were involved in the design and conduct of this research. During the grant-writing stage, priority of the research question and choice of outcome measures were informed by discussions with patients through HDRUK Breathe. The Northeast—Newcastle and North Tyneside 2 research ethics committee provided ethical approval for the CVD-COVID-UK/COVID-IMPACT research programme (REC No 20/NE/0161) to access, within secure trusted research environments, unconsented, whole-population, de-identified data from EHR data collected as part of patients’ routine health care.

## Supplementary Material

dyae068_Supplementary_Data

## Data Availability

The data used in this study are available in NHS England’s Secure Data Environment service for England, but as restrictions apply, they are not publicly available [https://digital.nhs.uk/services/secure-data-environment-service].
