# Supplementary material for: Risk of cardiovascular events following COVID-19 in people with and without pre-existing chronic respiratory disease
Source: Int J Epidemiol. 2024 Jun 7;53(3):dyae068. doi: 10.1093/ije/dyae068 (PMC11162089; doi:10.1093/ije/dyae068)
Supplement: dyae068_Supplementary_Data [file dyae068_supplementary_data.docx]

**Risk of cardiovascular disease in people with COVID-19 with pre-existing respiratory disease**

**Supplementary material**

Contents

[Additional methods 2](#_Toc163561584)

[Table S1: Data sources used in the English TRE 3](#_Toc163561585)

[Table S2: Number of cardiovascular outcome events during follow-up. 3](#_Toc163561586)

[Table S3: Baseline characteristics of people not excluded from the study compared with individuals included in the study 4](#_Toc163561587)

[Table S4: Cox regression estimates (hazard ratios) for the association between baseline chronic respiratory disease and risk of CV outcomes post-COVID-19 infection -unadjusted and fully adjusted for all tables 5](#_Toc163561588)

[Table S5: Cox regression estimates (hazard ratios) for the association between baseline chronic respiratory disease and risk of CV outcomes post-COVID-19 infection excluding people with a history of severe CV 6](#_Toc163561589)

[Table S6: Cox regression estimates (hazard ratios) for the association between baseline chronic respiratory disease and risk of CV outcomes post-COVID-19 infection within 30 days of follow-up. 7](#_Toc163561590)

[Table S7: Cox regression estimates (hazard ratios) for the association between baseline chronic respiratory disease and risk of CV outcomes post-COVID-19 infection in those who were hospitalised for COVID-19 8](#_Toc163561591)

[Table S8: Cox regression estimates (hazard ratios) for the association between baseline chronic respiratory disease and risk of CV outcomes post-COVID-19 infection in those who were not hospitalised for COVID-19 9](#_Toc163561592)

[Table S9: Adjusted Cox regression estimates (hazard ratios) for the association between increasing vaccine number and risk of CV outcomes post-COVID-19 infection in all patients, patients with pre-existing chronic respiratory disease, and patients with no pre-existing respiratory disease 9](#_Toc163561593)

[Table S10: Risk of CVD (and each CVD component) in COVID-19 patients with pre-existing asthma compared with COVID-19 patients without asthma 10](#_Toc163561594)

[Table S11: Risk of CV (and subgroup) following COVID-19 in people with asthma by asthma-related risk factors 11](#_Toc163561595)

[Table S12: Risk of CVD (and each CVD component) in COVID-19 patients with pre-existing COPD compared with COVID-19 patients without COPD 12](#_Toc163561596)

[Table S13: Risk of CV (and subgroup) following COVID-19 in people with COPD by COPD-related risk factors 13](#_Toc163561597)

[Figure S1: Study design. 14](#_Toc163561598)

[Figure S2: DAG for covariates in two example analyses. 15](#_Toc163561599)

# Additional methods

*Data quality checks*

Data quality checks were implemented to ensure data validity. Specifically, we ensured that: 1) date of birth was recorded before date of death; 2) recorded sex and date of birth were not missing; and 3) there were no recorded pregnancies or births for men and no recorded prostate cancer for women.

*Cardiovascular outcomes for the asthma and COPD cohorts*

For the asthma and COPD cohorts, these outcomes events were categorised into four variables: composite cardiovascular events, arterial events (disseminated intravascular coagulation-related events, stroke-related events, and myocardial infarction), venous events (venous thrombosis-related events and pulmonary embolism-related events), and other cardiovascular events (HF, angina, myocarditis, pericarditis, and arrhythmias) due to smaller population numbers.

*Baseline characteristics*

Baseline covariates were defined prior to the index date. These included age at index date, closest recorded body mass index (BMI) (categorised into underweight, normal, overweight, and obese), closest recorded Index of Multiple Deprivation score (in deciles), closest recorded smoking status (never, ex-smokers, current smokers), and history of hypertension, diabetes, liver disease, dementia, chronic kidney disease, and history of CVD, and ethnicity. Cardiovascular related medication, including antiplatelet, anti-lipid lowering, and anticoagulant medication, were defined as evidence of a prescription in the 3 months prior to the index date.

Fully adjusted models for the asthma specific analyses included: age, gender, smoking status, BMI, a history of hypertension, diabetes, liver disease, dementia, chronic kidney disease, cardiovascular disease (CVD), CVD-related medications 3 months prior to index date, region, index of multiple deprivation, ethnicity, short-acting bronchodilator use, exacerbations (for asthma control and ICS dose analysis), gastro-oesophageal reflux disease, and ICS dose (for exacerbation and asthma control analysis).

Fully adjusted models for the COPD specific analyses included: age, gender, smoking status, BMI, a history of hypertension, diabetes, liver disease, dementia, chronic kidney disease, cardiovascular disease (CVD), CVD-related medications 3 months prior to index date, region, index of multiple deprivation, ethnicity, short-acting bronchodilator use, height, exacerbations (for ICS analysis), gastro-oesophageal reflux disease, dyspnoea, ICS (for the exacerbation analysis), and COPD duration.

*Bonferroni*

Due to multiple testing, Bonferroni correction was applied whereby for each set of models. For the main analysis, a p-value threshold of 0.005 was used to define statistically significant results. This was determined by dividing the p-value threshold of 0.05 by 10 for the total number of outcomes (composite cardiovascular outcome and 9 individual cardiovascular outcomes) for each cohort of people investigated. A p-value threshold of 0.0125 was used to define statistically significant results for analyses in the asthma and COPD cohort. This was determined by dividing the p-value threshold of 0.05 by 4 for the total number of outcomes (composite cardiovascular disease, arterial events, venous events, other non-arterial or non-venous events).

# Table S1: Data sources used in the English TRE

| **Data source** | **Data source name and description** |
| --- | --- |
| Primary care data | General Practice Extraction Service (GPES) data for pandemic planning and research (GDPPR) |
| Hospital episodes data | Hospital Episode Statistics (HES) and Secondary Uses Service (SUS), including admitted patient care data, outpatient data, critical care data, and Emergency Care Dataset (ECDS) |
| Mortality data | Death registry data from the Office for National Statistics (ONS) |
| Community dispensing data | NHS Business Services Authority (BSA) community dispensing data |
| COVID-19 laboratory test data | Public Health England (PHE) Second Generation Surveillance System (SGSS) test data results for COVID-19 (pillars 1 and 2) |
| COVID-19 vaccination data | National immunisation management system COVID-19 vaccination dataset |

*Legend: TRE (trusted research environment)*

# Table S2: Number of cardiovascular outcome events during follow-up.

| **Cardiovascular outcome** | **n (%)** |
| --- | --- |
| Composite cardiovascular events | 43,820 (1.20) |
| Venous thromboembolic events | 2,030 (0.06) |
| Coagulopathy events | 725 (0.02) |
| Heart failure | 13,545 (0.37) |
| Angina | 5,300 (0.14) |
| Myocarditis and pericarditis | 185 (0.01) |
| Stroke | 11,615 (0.32) |
| Myocardial infarction | 5,440 (0.15) |
| Pulmonary and arterial embolism | 4,585 (0.12) |
| Arrythmias | 390 (0.01) |

*Legend: Venous thromboembolic events include deep vein and portal vein thrombosis. Coagulopathy events include thrombocytopenia, thrombophilia and mesenteric thrombus. Heart failure includes cardiomyopathy. All numbers are rounded to the nearest 5.*

# Table S3: Baseline characteristics of people not excluded from the study compared with individuals included in the study

| **Baseline covariate** | **Total cohort N=3,670,460** | **Population of included in study cohort N=2,483,685** |
| --- | --- | --- |
| **Age (mean, SD)** | 43.1 (18.4) | 47,1 (16.6) |
| **Female sex** | 1,982,115 (54.0) | 1,368,635 (55.1) |
| **Smoking status** Non-smoker  Ex-smoker  Current smoker  Missing | 2,150,610 (58.6) 833,550 (22.7) 534,220 (14.6) 152,075 (4.1) | 1,466,045 (59.0) 648,075 (26.1) 301,855 (12.2) 67,710 (2.7) |
| **IMD**  1 (most deprived)  2  3  4  5   6  7  8  9  10 (least deprived)  Missing | 468,930 (14.8) 455,030 (12.4) 433,325 (11.8) 391,800 (10.7) 363,685 (9.9) 341,140 (9.3) 325,870 (8.9) 317,555 (8.7) 296,420 (8.1) 260,590 (7.1) 16,115 (0.4) | 216,070 (8.7) 220,960 (8.9) 232,415 (9.4) 241,485 (9.7) 247,690 (10.0) 257,720 (10.4) 261,050 (10.5) 267,470 (10.8) 267,880 (10.8) 268,920 (10.8) 2,025 (0.08) |
| **Region** Southeast  Southwest  London  East Midlands   West Midlands  East of England  Yorkshire and The Humber  Northeast  Northwest  Missing | 455,290 (12.4) 181,690 (5.0)  638,670 (17.4) 269,940 (7.4) 477,860 (13.0) 305,840 (8.3) 394,475 (10.8)  162,560 (4.4) 580,275 (15.8) 203,855 (5.6) | 355,525 (14.3) 227,340 (9.2) 267,015 (10.8) 199,710 (8.0) 287,595 (11.6) 211,990 (8.5) 288,400 (11.6)  125,255 (5.0) 352,290 (14.2) 168,575 (6.8) |
| **BMI**  Underweight  Normal   Overweight  Obese  Missing | 120,075 (3.3)  902,700 (24.6) 791,070 (21.6)  1,417,310 (38.6)  439,305 (12.0) | 57,825 (2.3) 614,585 (24.8) 570,760 (23.0) 1,028,785 (41.4) 211,730 (8.5) |
| **Hypertension** | 754,915 (20.6) | 526,535 (21.2) |
| **Diabetes** | 320,765 (8.7) | 217,375 (8.8) |
| **Liver disease** | 12,185 (0.3) | 6,425 (0.3) |
| **Chronic kidney disease** | 252,660 (6.9) | 138,490 (5.6) |
| **Dementia** | 101,130 (2.8) | 34,950 (1.4) |
| **Previous severe cardiovascular** **events** | 344,445 (9.4) | 210,465 (8.5) |
| **Cardiovascular-related medication** | 797,235 (21.7) | 584,980 (23.6) |

# Table S4: Cox regression estimates (hazard ratios) for the association between baseline chronic respiratory disease and risk of CV outcomes post-COVID-19 infection -unadjusted and fully adjusted for all tables

|  | Number of CV events (n, %) | | Absolute risks | | Cox regression estimates | | | |
| --- | --- | --- | --- | --- | --- | --- | --- | --- |
| CV outcome | Pre-existing respiratory disease | No pre-existing respiratory disease | Pre-existing respiratory disease (per 100,000 person-weeks) | No pre-existing respiratory disease (per 100,000 person-weeks | Crude HR (95% CI) | P value | Adjusted HR (95% CI) | P value |
| Composite CV | 11,765 (1.76) | 32,055 (1.07) | 92.6 (91.0-94.3) | 52.5 (52.0-53.1) | 1.71 (1.67-1.75) | <0.0001* | 1.08 (1.06-1.11) | <0.0001* |
| VTE | 490 (0.07) | 1,545 (0.05) | 3.8 (3.5-4.2) | 2.5 (2.4-2.6) | 1.48 (1.34-1.63) | <0.0001* | 1.16 (1.05-1.29) | 0.0056 |
| Thrombo | 190 (0.03) | 535 (0.02) | 1.5 (1.3-1.7) | 0.9 (0.8-1.0) | 1.63 (1.37-1.93) | <0.0001* | 1.11 (0.92-1.33) | 0.2644 |
| HF | 4,200 (0.63) | 9,345 (0.31) | 32.8 (31.8-33.8) | 15.2 (14.9-15.5) | 2.10 (2.02-2.18) | <0.0001* | 1.17 (1.12-1.22) | <0.0001* |
| Angina | 1,500 (0.22) | 3,800 (0.13) | 11.7 (11.1-12.3) | 6.2 (6.0-6.4) | 1.85 (1.74-1.97) | <0.0001* | 1.13 (1.06-1.20) | 0.0003* |
| Myo/pericarditis | 35 (0.01) | 145 (0.00) | 0.3 (0.2-0.4) | 0.2 (0.2-0.3) | 1.19 (0.82-1.72) | 0.354 | 1.15 (0.78-1.69) | 0.4840 |
| Stroke | 2,665 (0.40) | 8,950 (0.30) | 20.8 (20.0-21.6) | 14.6 (14.3-14.9) | 1.38 (1.32-1.44) | <0.0001* | 0.89 (0.85-0.94) | <0.0001* |
| MI | 1,450 (0.22) | 3,990 (0.13) | 11.3 (10.7-11.9) | 6.5 (6.3-6.7) | 1.68 (1.58-1.78) | <0.0001* | 1.06 (1.00-1.13) | 0.0600 |
| PE | 1,130 (0.17) | 3,455 (0.12) | 8.8 (8.3-9.3) | 5.6 (8.3-9.3) | 1.50 (1.41-1.61) | <0.0001* | 1.24 (1.15-1.33) | <0.0001* |
| Arrythmia | 105 (0.02) | 285 (0.01) | 0.8 (0.7-1.0) | 0.4 (0.4-0.5) | 1.75 (1.39-2.20) | <0.0001* | 1.12 (0.88-1.43) | 0.3518 |

*Legend: Venous thromboembolic events include deep vein and portal vein thrombosis. Coagulopathy events include thrombocytopenia, thrombophilia and mesenteric thrombus. CV (cardiovascular), PE (pulmonary embolism).*Due to multiple testing, Bonferroni correction was applied whereby for each set of models, a p-value threshold of 0.005 was used.*

#

# Table S5: Cox regression estimates (hazard ratios) for the association between baseline chronic respiratory disease and risk of CV outcomes post-COVID-19 infection excluding people with a history of severe CV

|  | Number of CV events (n, %) | |  |  |  |  |
| --- | --- | --- | --- | --- | --- | --- |
| CV outcome | Pre-existing respiratory disease | No pre-existing respiratory disease | Crude HR (95% CI) | P value | Adjusted HR (95% CI) | P value |
| Composite CV | 5,990 (0.98) | 17,830 (0.63) | 1.61 (1.56-1.66) | <0.0001* | 1.13 (1.09-1.16) | <0.0001* |
| VTE | 340 (0.06) | 1,145 (0.04) | 1.42 (1.26-1.60) | <0.0001* | 1.23 (1.08-1.40) | 0.0015* |
| Thrombo | 125 (0.02) | 395 (0.01) | 1.53 (1.25-1.88) | <0.0001* | 1.12 (0.90-1.40) | 0.3190 |
| HF | 2,190 (0.36) | 5,200 (0.18) | 2.02 (1.92-2.13) | <0.0001* | 1.15 (1.09-1.22) | <0.0001* |
| Angina | 725 (0.12) | 2,040 (0.07) | 1.72 (1.58-1.87) | <0.0001* | 1.10 (1.00-1.21) | 0.0393 |
| Myo/pericarditis | 30 (0.00) | 135 (0.00) | 1.08 (0.73-1.60) | 0.6858 | 1.02 (0.68-1.55) | 0.9071 |
| Stroke | 1,030 (0.17) | 3,900 (0.14) | 1.26 (1.18-1.35) | <0.0001* | 0.98 (0.91-1.05) | 0.5233 |
| MI | 625 (0.10) | 1,960 (0.07) | 1.52 (1.38-1.66) | <0.0001* | 1.14 (1.03-1.25) | 0.0083 |
| PE | 865 (0.14) | 2,875 (0.10) | 1.43 (1.32-1.54) | <0.0001* | 1.24 (1.14-1.34) | <0.0001* |
| Arrythmia | 60 (0.01) | 180 (0.01) | 1.64 (1.22-2.20) | 0.0011* | 1.15 (0.84-1.58) | 0.3883 |

*Legend: Venous thromboembolic events include deep vein and portal vein thrombosis. Coagulopathy events include thrombocytopenia, thrombophilia and mesenteric thrombus. CV (cardiovascular), PE (pulmonary embolism). * Due to multiple testing, Bonferroni correction was applied whereby for each set of models, a p-value threshold of 0.005 was used.*

#

# Table S6: Cox regression estimates (hazard ratios) for the association between baseline chronic respiratory disease and risk of CV outcomes post-COVID-19 infection within 30 days of follow-up.

|  | **Total population** | | | | **No history of severe CV disease** | |
| --- | --- | --- | --- | --- | --- | --- |
| CV outcome | Unadjusted HR  (95% CI) | P value | Adjusted HR  (95% CI) | P value | Adjusted HR  (95% CI) | P value |
| Composite CV | 1.60 (1.55-1.65) | <0.0001 | 1.05 (1.02-1.09) | 0.0046* | 1.10 (1.05-1.16) | <0.0001* |
| VTE | 1.29 (1.10-1.50) | 0.0013 | 1.10 (0.93-1.29) | 0.2647 | 1.15 (0.95-1.39) | 0.1459 |
| Thrombo | 1.49 (1.18-1.89) | 0.0010 | 1.09 (0.84-1.40) | 0.5207 | 1.21 (0.90-1.63) | 0.2151 |
| HF | 1.99 (1.89-2.10) | <0.0001 | 1.12 (1.06-1.19) | 0.0001* | 1.07 (0.99-1.16) | 0.0930 |
| Angina | 1.84 (1.67-2.02) | <0.0001 | 1.15 (1.04-1.28) | 0.0055 | 1.10 (0.95-1.28) | 0.2116 |
| Myo/pericarditis | 1.21 (0.69-2.15) | 0.5059 | 1.14 (0.62-2.09) | 0.6699 | 0.82 (0.40-1.68) | 0.5874 |
| Stroke | 1.30 (1.22-1.38) | <0.0001 | 0.87 (0.81-0.92) | <0.0001* | 0.96 (0.87-1.06) | 0.4031 |
| MI | 1.62 (1.50-1.76) | <0.0001 | 1.05 (0.97-1.14) | 0.2227 | 1.20 (1.0-1.35) | 0.0036* |
| PE | 1.41 (1.29-1.54) | <0.0001 | 1.23 (1.12-1.34) | <0.0001* | 1.23 (1.11-1.37) | 0.0001* |
| Arrythmia | 1.68 (1.24-2.29) | 0.0010 | 1.14 (0.83-1.57) | 0.4222 | 1.27 (0.84-1.91) | 0.2559 |

*Legend: Venous thromboembolic events include deep vein and portal vein thrombosis. Coagulopathy events include thrombocytopenia, thrombophilia and mesenteric thrombus. CV (cardiovascular), PE (pulmonary embolism). * Due to multiple testing, Bonferroni correction was applied whereby for each set of models, a p-value threshold of 0.005 was used.*

#

# Table S7: Cox regression estimates (hazard ratios) for the association between baseline chronic respiratory disease and risk of CV outcomes post-COVID-19 infection in those who were hospitalised for COVID-19

|  | **Complete population** | | **No history of severe CV disease** | |
| --- | --- | --- | --- | --- |
| CV outcome | Adjusted HR (95% CI) | P value | Adjusted HR (95% CI) | P value |
| Composite CV | 0.95 (0.91-0.98) | 0.0056 | 0.93 (0.88-0.98) | 0.0119 |
| VTE | 0.87 (0.71-1.06) | 0.1657 | 0.95 (0.75-1.21) | 0.6999 |
| Thrombo | 0.76 (0.55-1.06) | 0.1051 | 0.71 (0.47-1.08) | 0.1131 |
| HF | 1.08 (1.01-1.14) | 0.0198 | 1.00 (0.92-1.09) | 0.9823 |
| Angina | 1.00 (0.88-1.12) | 0.9402 | 0.95 (0.79-1.14) | 0.5735 |
| Myo/pericarditis | 1.04 (0.45-2.41) | 0.9194 | 0.99 (0.42-2.35) | 0.9834 |
| Stroke | 0.76 (0.70-0.82) | <0.0001* | 0.81 (0.71-0.92) | 0.0013* |
| MI | 0.98 (0.87-1.09) | 0.6597 | 1.00 (0.84-1.18) | 0.9566 |
| PE | 0.99 (0.88-1.11) | 0.8481 | 1.03 (0.90-1.17) | 0.6798 |
| Arrythmia | 0.82 (0.52-1.30) | 0.3990 | 0.51 (0.26-0.99) | 0.0474 |

*Legend: Venous thromboembolic events include deep vein and portal vein thrombosis. Coagulopathy events include thrombocytopenia, thrombophilia and mesenteric thrombus. CV (cardiovascular), PE (pulmonary embolism). * Due to multiple testing, Bonferroni correction was applied whereby for each set of models, a p-value threshold of 0.005 was used.*

#

# Table S8: Cox regression estimates (hazard ratios) for the association between baseline chronic respiratory disease and risk of CV outcomes post-COVID-19 infection in those who were not hospitalised for COVID-19

|  | **Total population** | | **No history of severe CV disease** | |
| --- | --- | --- | --- | --- |
| CV outcome | Adjusted HR (95% CI) | P value | Adjusted HR (95% CI) | P value |
| Composite CV | 1.11 (1.08-1.14) | <0.0001* | 1.16 (1.11-1.21) | <0.0001* |
| VTE | 1.20 (1.06-1.36) | 0.0037* | 1.24 (1.07-1.44) | 0.0051 |
| Thrombo | 1.23 (1.00-1.53) | 0.0555 | 1.24 (0.96-1.60) | 0.0955 |
| HF | 1.19 (1.13-1.25) | <0.0001* | 1.20 (1.12-1.29) | <0.0001* |
| Angina | 1.14 (1.06-1.23) | 0.0006* | 1.11 (1.00-1.23) | 0.0479 |
| Myo/pericarditis | 1.10 (0.71-1.71) | 0.6673 | 0.96 (0.60-1.53) | 0.8518 |
| Stroke | 0.94 (0.89-0.99) | 0.0191 | 1.00 (0.92-1.09) | 0.9788 |
| MI | 1.07 (0.99-1.16) | 0.0722 | 1.15 (1.02-1.29) | 0.0222 |
| PE | 1.24 (1.14-1.36) | <0.0001* | 1.20 (1.08-1.33) | 0.0005* |
| Arrythmia | 1.24 (0.94-1.64) | 0.1278 | 1.43 (1.01-2.04) | 0.0440 |

*Legend: Venous thromboembolic events include deep vein and portal vein thrombosis. Coagulopathy events include thrombocytopenia, thrombophilia and mesenteric thrombus. CV (cardiovascular), PE (pulmonary embolism). *Due to multiple testing, Bonferroni correction was applied whereby for each set of models, a p-value threshold of 0.005 was used.*

# Table S9: Adjusted Cox regression estimates (hazard ratios) for the association between increasing vaccine number and risk of CV outcomes post-COVID-19 infection in all patients, patients with pre-existing chronic respiratory disease, and patients with no pre-existing respiratory disease

|  | All patients | | Patients with pre-existing respiratory disease | | Patients with no pre-existing respiratory disease | |
| --- | --- | --- | --- | --- | --- | --- |
| Composite CV | Adjusted HR (95% CI) | P value | Adjusted HR (95% CI) | P value | Adjusted HR (95% CI) | P value |
| Unvaccinated period | Reference |  | Reference |  | Reference |  |
| Dose 1 | 0.56 (0.55-0.56) | <0.0001 | 0.59 (0.56-0.61) | <0.0001 | 0.56 (0.55-0.58) | <0.0001 |
| Dose 2 | 0.55 (0.54-0.56) | <0.0001 | 0.58 (0.56-0.59) | <0.0001 | 0.55 (0.54-0.56) | <0.0001 |
| Booster | 0.30 (0.28-0.32) | <0.0001 | 0.30 (0.28-0.33) | <0.0001 | 0.30 (0.28-0.32) | <0.0001 |

*Legend: Reference period is the unvaccinated period from covid-19 infection to first covid-19 vaccination or censoring. The dose 1 period is from 1^st^ covid-19 vaccination to 2^nd^ covid-19 vaccination or censoring. The dose 2 period is from the 2^nd^ covid-19 vaccination to booster or censoring. The booster period is from the booster vaccine to end of follow-up or censoring. CV (cardiovascular).*

# Table S10: Risk of CVD (and each CVD component) in COVID-19 patients with pre-existing asthma compared with COVID-19 patients without asthma

|  | **Complete population** | | | | **No history of severe CV disease** | | | |
| --- | --- | --- | --- | --- | --- | --- | --- | --- |
| CV outcome | Unadjusted HR (95% CI) | P value | Adjusted HR (95% CI) | P value | Unadjusted HR (95% CI) | P value | Adjusted HR (95% CI) | P value |
| Composite CV | 1.10 (1.07-1.13) | <0.0001 | 1.05 (1.02-1.08) | 0.0002* | 1.10 (1.06-1.14) | <0.0001 | 1.09 (1.05-1.13) | <0.0001* |
| VTE | 1.20 (1.08-1.34) | 0.0009 | 1.18 (1.05-1.32) | 0.0052 | 1.20 (1.06-1.36) | 0.0050 | 1.23 (1.07-1.40) | 0.0030* |
| Thrombo | 1.07 (0.87-1.30) | 0.5299 | 1.00 (0.82-1.24) | 0.9657 | 1.08 (0.86-1.36) | 0.5216 | 1.06 (0.83-1.35) | 0.6497 |
| HF | 1.18 (1.13-1.24) | <0.0001 | 1.08 (1.04-1.13) | 0.0004* | 1.19 (1.12-1.27) | <0.0001 | 1.09 (1.02-1.16) | 0.0099 |
| Angina | 1.24 (1.16-1.33) | <0.0001 | 1.11 (1.03-1.20) | 0.0040* | 1.26 (1.15-1.39) | <0.0001 | 1.13 (1.02-1.25) | 0.0162 |
| Myo/pericarditis | 1.09 (0.74-1.60) | 0.6544 | 1.17 (0.78-1.75) | 0.4545 | 1.02 (0.68-1.54) | 0.9191 | 1.06 (0.69-1.63) | 0.7869 |
| Stroke | 0.91 (0.87-0.96) | 0.0003 | 0.90 (0.86-0.95) | 0.0002* | 0.85 (0.79-0.92) | 0.0001 | 0.92 (0.84-1.00) | 0.0402 |
| MI | 1.10 (1.03-1.18) | 0.0050 | 1.07 (1.00-1.15) | 0.0564 | 1.04 (0.94-1.16) | 0.4208 | 1.12 (1.00-1.24) | 0.0477 |
| PE | 1.12 (1.03-1.20) | 0.0043 | 1.16 (1.07-1.26) | 0.0002* | 1.13 (1.04-1.22) | 0.0055 | 1.18 (1.08-1.29) | 0.0002* |
| Arrythmia | 1.26 (0.97-1.62) | 0.0787 | 1.16 (0.89-1.52) | 0.2773 | 1.30 (0.95-1.78) | 0.1031 | 1.20 (0.86-1.67) | 0.2790 |

*Legend: Venous thromboembolic events include deep vein and portal vein thrombosis. Coagulopathy events include thrombocytopenia, thrombophilia and mesenteric thrombus. CV (cardiovascular), PE (pulmonary embolism). *Due to multiple testing, Bonferroni correction was applied whereby for each set of models, a p-value threshold of 0.005 was used.*

#

# Table S11: Risk of CV (and subgroup) following COVID-19 in people with asthma by asthma-related risk factors

| **CV outcome** | | **Complete population** | | | | **No history of severe CV disease** | | | |
| --- | --- | --- | --- | --- | --- | --- | --- | --- | --- |
|  |  | Unadjusted HR (95% CI) | P value | Adjusted HR (95% CI) | P value | Unadjusted HR (95% CI) | P value | Adjusted HR (95% CI) | P value |
| **Asthma control** | **Composite CV** | 5.33 (5.09-5.59) | <0.0001* | 1.13 (1.05-1.22) | 0.0008* | 5.85 (5.51-6.21) | <0.0001* | 1.13 (1.03-1.24) | 0.0082 |
|  | **Arterial events** | 4.93 (4.57-5.31) | <0.0001* | 1.16 (1.02-1.31) | 0.0194 | 3.91 (2.75-5.56) | <0.0001* | 1.16 (0.60-2.23) | 0.6585 |
|  | **Venous events** | 3.24 (2.88-3.63) | <0.0001* | 1.15 (0.96-1.38) | 0.1291 | 3.49 (3.04-4.01) | <0.0001* | 1.22 (0.99-1.51) | 0.0608 |
|  | **Other CV events** | 6.56 (6.13-7.02) | <0.0001* | 1.11 (1.00-1.23) | 0.0442 | 6.64 (6.21-7.11) | <0.0001* | 1.11 (1.00-1.23) | 0.0414 |
| **Asthma exacerbations** | **Composite CV** | 4.81 (4.58-5.05) | <0.0001* | 1.36 (1.27-1.46) | <0.0001* | 5.13 (4.82-5.46) | <0.0001* | 1.38 (1.26-1.51) | <0.0001* |
|  | **Arterial events** | 4.59 (4.24-4.96) | <0.0001* | 1.36 (1.21-1.53) | <0.0001* | 4.08 (2.80-5.93) | <0.0001* | 1.36 (0.74-2.53) | 0.3254 |
|  | **Venous events** | 2.97 (2.60-3.38) | <0.0001* | 1.18 (0.98-1.43) | 0.0833 | 3.05 (2.61-3.58) | <0.0001* | 1.20 (0.96-1.50) | 0.1067 |
|  | **Other CV events** | 5.64 (5.25-6.05) | <0.0001* | 1.39 (1.26-1.54) | <0.0001* | 5.72 (5.33-6.14) | <0.0001* | 1.42 (1.29-1.57) | <0.0001* |
| **ICS dose** | **Composite CV**  Low dose   Medium dose  High dose | Reference  1.59 (1.49-1.69) 2.00 (1.81-2.22) | <0.0001* <0.0001* | Reference  1.08 (1.04-1.16) 1.06 (0.96-1.18) | 0.0182 0.2475 | Reference  1.64 (1.51-1.78) 2.31 (2.04-2.62) | <0.0001* <0.0001* | Reference  1.09 (1.00-1.18) 1.19 (1.04-1.35) | 0.0604 0.0095 |
|  | **Arterial events**   Low dose   Medium dose  High dose | Reference  1.47 (1.31-1.64) 1.53 (1.27-1.84) | <0.0001* <0.0001* | Reference  1.03 (0.92-1.15) 0.81 (0.67-0.99) | 0.6345 0.0389 | Reference 1.18 (0.68-2.06) 1.09 (0.39-3.06) | 0.5530 0.8642 | Reference  0.75 (0.42-1.35) 0.60 (0.21-1.71) | 0.3420 0.3349 |
|  | **Venous events**   Low dose   Medium dose  High dose | Reference  1.56 (1.33-1.84) 1.68 (1.28-2.22) | <0.0001* 0.0002* | Reference  1.23 (1.03-1.46) 1.05 (0.78-1.41) | 0.0190 0.7355 | Reference 1.53 (1.25-1.86) 1.84 (1.33-2.55) | <0.0001* 0.0002* | Reference  1.17 (0.95-1.43) 1.09 (0.78-1.54) | 0.1432 0.6153 |
|  | **Other CV events**  Low dose   Medium dose  High dose | Reference  1.67 (1.52-1.83) 2.44 (2.13-2.80) | <0.0001* <0.0001* | Reference  1.08 (0.98-1.19) 1.24 (1.08-1.43) | 0.1224 0.0026* | Reference  1.68 (1.53-1.84) 2.45 (2.14-2.81) | <0.0001* <0.0001* | Reference  1.08 (0.98-1.19) 1.22 (1.06-1.41) | 0.1203 0.0054 |

*Legend: *Due to multiple testing, Bonferroni correction was applied whereby for each set of models, a p-value threshold of 0.0125 was used.*

#

# Table S12: Risk of CVD (and each CVD component) in COVID-19 patients with pre-existing COPD compared with COVID-19 patients without COPD

|  | **Complete population** | | | | **No history of severe CV disease** | | | |
| --- | --- | --- | --- | --- | --- | --- | --- | --- |
| CV outcome | Unadjusted HR (95% CI) | P value | Adjusted HR (95% CI) | P value | Unadjusted HR (95% CI) | P value | Adjusted HR (95% CI) | P value |
| Composite CV | 7.81 (7.51-8.12) | <0.0001* | 1.11 (1.07-1.14) | <0.0001* | 8.44 (8.03-8.88) | <0.0001* | 1.18 (1.13-1.24) | <0.0001* |
| VTE | 4.15 (3.48-4.93) | <0.0001* | 1.08 (0.89-1.30) | 0.4456 | 4.37 (3.49-5.46) | <0.0001* | 1.20 (0.95-1.53) | 0.1263 |
| Thrombo | 6.71 (5.30-8.49) | <0.0001* | 1.26 (0.97-1.64) | 0.0789 | 7.54 (5.57-10.22) | <0.0001* | 1.33 (0.95-1.88) | 0.0967 |
| HF | 11.04 (10.45-11.65) | <0.0001* | 1.25 (1.18-1.31) | <0.0001* | 13.08 (12.15-14.07) | <0.0001* | 1.22 (1.14-1.32) | <0.0001* |
| Angina | 8.03 (7.36-8.76) | <0.0001* | 1.14 (1.04-1.24) | 0.0046* | 7.66 (6.70-8.76) | <0.0001* | 0.98 (0.85-1.14) | 0.8195 |
| Myo/pericarditis | 2.26 (1.07-4.79) | 0.0336 | 1.13 (0.48-2.68) | 0.7839 | 1.96 (0.73-5.31) | 0.1845 | 0.83 (0.25-2.77) | 0.7612 |
| Stroke | 6.00 (5.62-6.40) | <0.0001* | 0.87 (0.81-0.93) | 0.0001* | 6.70 (6.04-7.43) | <0.0001* | 1.08 (0.96-1.21) | 0.1807 |
| MI | 7.06 (6.47-7.70) | <0.0001* | 1.03 (0.94-1.13) | 0.4976 | 7.78 (6.78-8.93) | <0.0001* | 1.21 (1.05-1.40) | 0.0098 |
| PE | 4.96 (4.45-5.53) | <0.0001* | 1.36 (1.21-1.52) | <0.0001* | 5.03 (4.38-5.77) | <0.0001* | 1.30 (1.12-1.51) | 0.0004* |
| Arrythmia | 8.52 (6.39-11.36) | <0.0001* | 1.46 (1.05-2.03) | 0.0263 | 7.67 (4.98-11.83) | <0.0001* | 1.33 (0.80-2.20) | 0.2664 |

*Legend: Venous thromboembolic events include deep vein and portal vein thrombosis. Coagulopathy events include thrombocytopenia, thrombophilia and mesenteric thrombus. CV (cardiovascular), PE (pulmonary embolism). *Due to multiple testing, Bonferroni correction was applied whereby for each set of models, a p-value threshold of 0.005 was used.*

#

# Table S13: Risk of CV (and subgroup) following COVID-19 in people with COPD by COPD-related risk factors

| **CV outcome** | | **Complete population** | | | | **No history of severe CV disease** | | | |
| --- | --- | --- | --- | --- | --- | --- | --- | --- | --- |
|  |  | Unadjusted HR (95% CI) | P value | Adjusted HR (95% CI) | P value | Unadjusted HR (95% CI) | P value | Adjusted HR (95% CI) | P value |
| **ICS use** | **Composite CV** | 1.11 (1.04-1.19) | 0.0018* | 0.93 (0.86-1.00) | 0.0593 | 1.20 (1.11-1.30) | <0.0001* | 0.98 (0.89-1.07) | 0.6369 |
|  | **Arterial events** | 0.97 (0.88-1.07) | 0.5294 | 0.86 (0.75-0.97) | 0.0158 | 1.06 (0.67-1.65) | 0.8101 | 0.77 (0.39-1.51) | 0.4430 |
|  | **Venous events** | 1.22 (1.03-1.45) | 0.0236 | 1.06 (0.83-1.36) | 0.6316 | 1.29 (1.06-1.58) | 0.0133 | 1.18 (0.88-1.57) | 0.2717 |
|  | **Other CV events** | 1.20 (1.10-1.30) | <0.0001* | 0.97 (0.57-1.37) | 0.5301 | 1.19 (1.10-1.30) | <0.0001* | 0.96 (0.87-1.07) | 0.4575 |
| **COPD exacerbations** | **Composite CV** | 2.24 (2.09-2.39) | <0.0001* | 1.35 (1.24-1.46) | <0.0001* | 2.49 (2.29-2.70) | <0.0001* | 1.42 (1.27-1.58) | <0.0001* |
|  | **Arterial events** | 1.89 (1.71-2.08) | <0.0001* | 1.21 (1.06-1.39) | 0.0049* | 2.07 (1.31-3.29) | 0.0020* | 1.01 (0.52-1.97) | 0.9677 |
|  | **Venous events** | 1.56 (1.30-1.86) | <0.0001* | 1.28 (0.98-1.66) | 0.0654 | 1.52 (1.24-1.86) | <0.0001* | 1.19 (0.87-1.62) | 0.2870 |
|  | **Other CV events** | 2.62 (2.40-2.86) | <0.0001* | 1.44 (1.28-1.62) | <0.0001* | 2.68 (2.45-2.92) | <0.0001* | 1.47 (1.30-1.65) | <0.0001* |

*Legend: *Due to multiple testing, Bonferroni correction was applied whereby for each set of models, a p-value threshold of 0.0125 was used.*


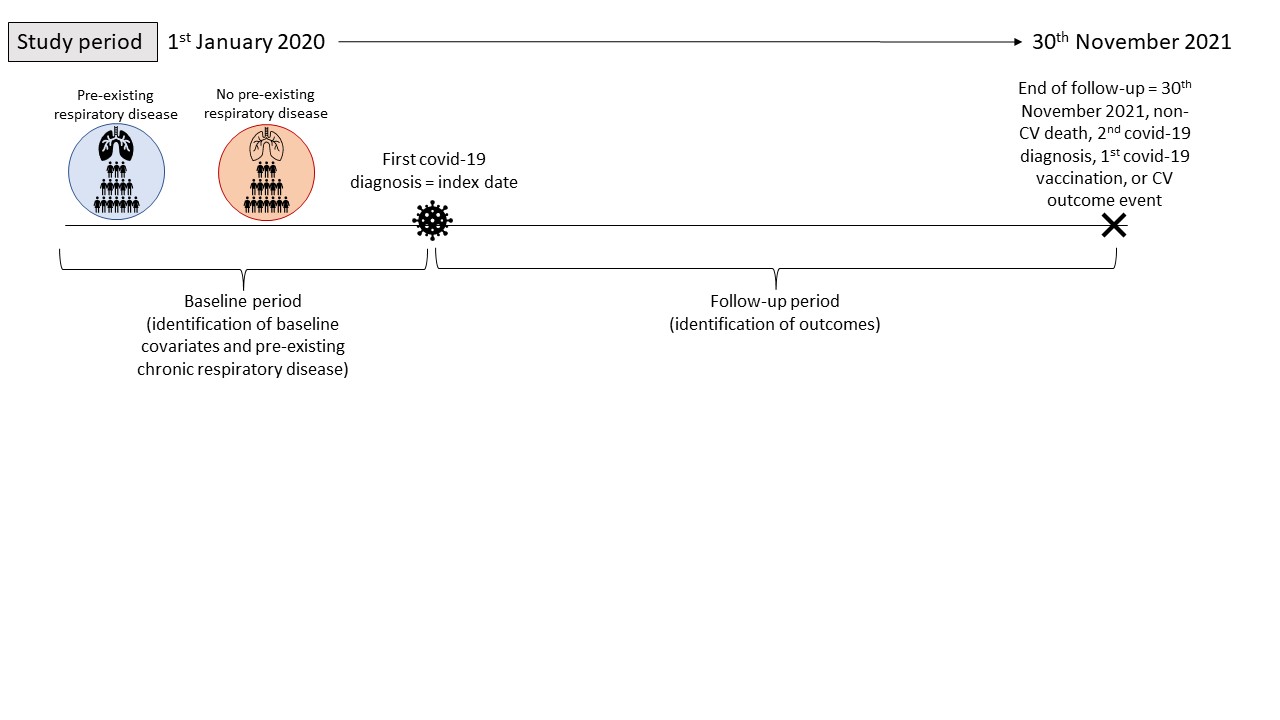


# Figure S1: Study design.

*Legend: Pre-existing respiratory disease is defined as a diagnosis of chronic obstructive pulmonary disease, asthma, bronchiectasis, cystic-fibrosis, or interstitial lung disease. CV (cardiovascular)*


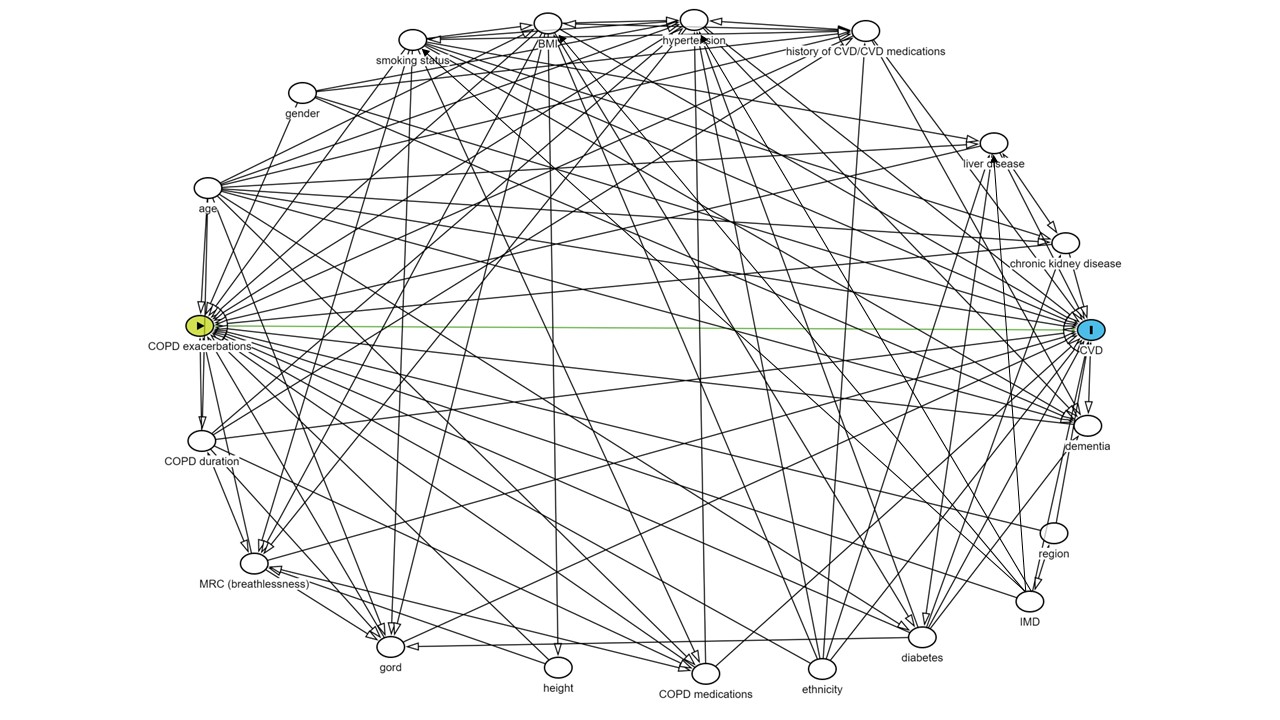

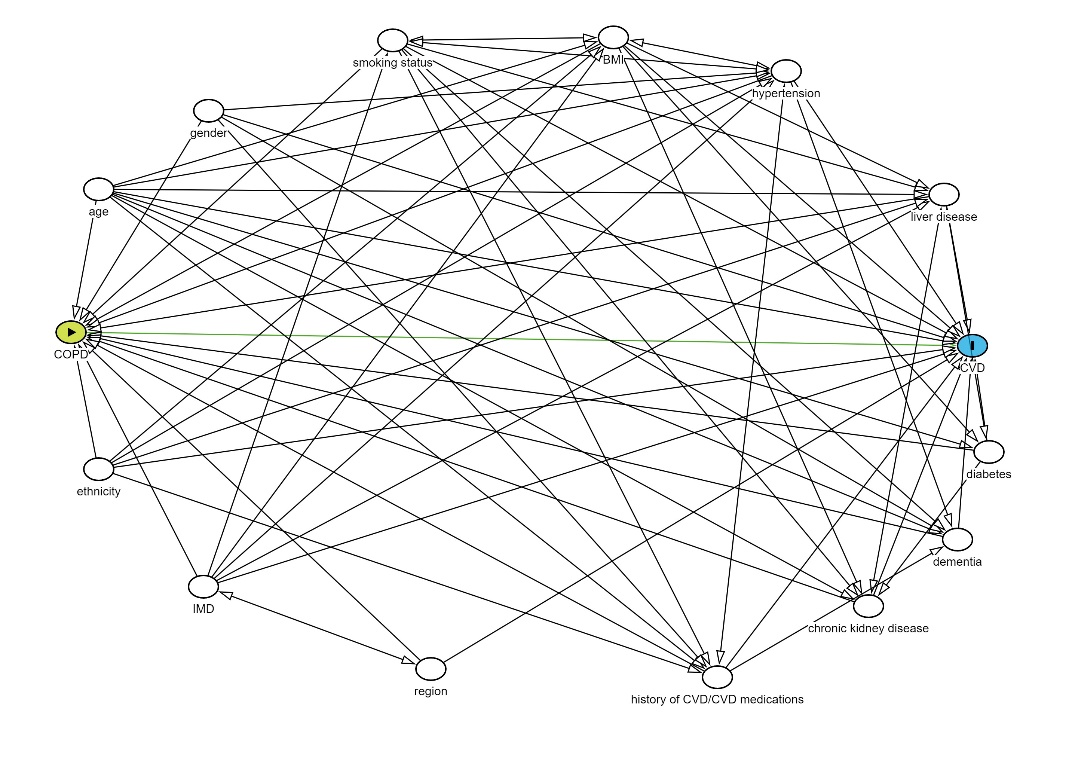


# Figure S2: DAG for covariates in two example analyses.

*Legend: A) Covariates used in analysis investigating pre-existing COPD and risk of CVD following COVID-19 and B) Covariates used in analysis investigating COPD exacerbations and risk of CVD following COVID-19. Covariates used in (A) are also used in all main analyses. Covariates used in (B) are also used in the COPD ICS analysis (including adjusting for exacerbations of COPD).*
